# Philadelphia Hospital

**Published:** 1838-02-14

**Authors:** 


### PHILADELPHIA HOSPITAL.

### NEURALGIA.

(*Concluded from page* 55.)

*Saturday, Jan. 6th.*—(Dr. Jackson continued.) Some patients will now be introduced illustrative of this form of disease.

About two months since, this woman had a severe attack of pain in the ulnar nerve. You perceive, that pressure on the lumbar spine, produces great uneasiness in the hypogastric region. The pain, from the account she gives, is not constant; it is fluctuating. This latter circumstance may be called one of the distinctive characters of the disease. From this patient's suffering under another affection, the neuralgic disease is not so well marked. She is labouring under an uterine disease, and in females it is singular that the neuralgia are almost always accompanied by some disorder of the womb.

Here is another patient. This young woman has been sick since last May. She was first attacked with acute pain in the head. The sensibility of the whole of one half of the body is, at this time, morbidly developed. The slightest touch, as you see, produces the most exquisite pain down the whole of that side of the body. The skin is usually dry and cold; in this case it is moist; this is owing to the sweating of hectic fever, under which this girl labors. In the course of the disease phthisis has been developed, and a cavity exists in the left lung. In this patient you see sensibility inordinately developed throughout one half of the body. Touch any portion of the left side of this girl, and you produce infinite torture. The right side offers a different state. There the sensation is natural; you find she does not shrink from the approach of the hand. Sensation continues normal, neither augmented, nor decreased. This is an illustration of what was said of the arrangement of the nervous system. If sensibility were an unit, this partial affection could not occur. You see as this patient is carried from the room, that the very motion of the bed produces great pain. At one period of the disease she could not bear even the contact of the bed clothes. In this case the uterine function preserves its integrity, but previous to the attack she suffered from considerable derangement of the digestive organs, arising from a sedentary employment. Here is another case. In this woman the two lateral halves of the body are in opposite conditions. The left side has its sensibility morbidly increased. You see that she winces from a slight touch. The right side is destitute of feeling—there is palsy of sensibility—*anœthesia.* You perceive that she does not feel the pricking of a pin. She is unconscious that it is plunged into the skin. The median line divides these two states. Even in the tongue this is palpably evident. In the left side there is feeling; she shrinks from the prick of a pin; the right side gives no perception of the wound. The sense of taste too is absent in the right side. This case exhibits another remarkable feature. I press now on the upper cervical vertibræ, and she informs you that she feels pain in her neck, and in the lower part of the face. I make pressure now between the shoulders, and she tells you that she has pain in the breast. The pressure is now made in the middle of the back, and she experiences a painful sensation in the hypochondriac and epigastric regions. At times, if the pressure there be very forcible, she suffers nausea, and even vomiting has been excited. I now press on the lumbar vertebraæ, and she suffers from pain in the hypogastric region, and in the bowels. This patient is laboring under complicated uterine diseases.

These cases are beautiful illustrations of the pathology of the nervous system, and this last exemplifies the doctrine of segmentation of the spinal marrow, with the presidence of the segment over corresponding regions of the body. You see that she staggers in her walk; the want of sensibility in the foot causes this imperfect progression. In the absence of sensibility the eye directs our movements, and when it is taken away from the ground, in such instances the limb gives way. The muscles loose their tension from want of the reflected action of sensibility. Thus in walking we have an illustration of the reflex action.

I present to you another case. At this time the patient does not complain of pain, but press on any part of the spine, and immediately she suffers acute pain all over. In this case also the neuralgia is complicated with uterine disease. She had prolapsus anterior to the commencement of the nervous disorder. In all these cases, with the exception to which I called your attention, the skin is cold, dry and harsh.

You have now seen cases which illustrate very well the character of neuralgia; the perversion, and exaltation of nervous sensibility. Sometimes it occupies the whole body; sometimes it is limited to one half, sometimes so purely localized that you may cover the seat of the pain, with the point of a pin, yet, still, so very acute in its nature, as to be intolerable from its sharpness. Sometimes it involves only an inch to an inch and a half of the nerve; sometimes it is fixed to one spot; sometimes it wanders, attacking in succession every part of the body. We have it under every variety of character, from a general and diffused pain, down to the minutest point; we have it flying about, attacking part after part, organ after organ, and we have it stationary.

So far we have been engaged in the consideration of the disorders of external sensibility alone. But we have also other classes of the disease. The internal organs, are likewise effected with neuralgic pains, constituting the visceral neuralgia. All the organs may present this modification of their sensibility. It must be distinguished from the inflammations of the same organs.

A different nomenclature is employed to designate the two. The termination *itis* denotes the inflammation, and the termination *algia* designates the nervous pain of the organ. Thus we have gastritis and gastralgia, hepatitis and hepatalgia, enteritis and enteralgia, cystitis and cystalgia, pleuritis and pleuralgia, &c. &c.

The diagnosis of inflammations and of nervous pains is exceedingly important, especially in the internal affections. It was formerly supposed that pain was always a product of inflammation, and that pain was a certain indication of its existence. We now know better. When the neuralgic affection is external the absence of redness, of swelling, and of heat in the seat of pain readily enables us to ascertain that the affection is not inflammatory. But we cannot have the same evidence in the internal disorders. Internal inflammations producing the same intensity of pain, as occurs in an internal neuralgia, would inevitably occasion fever. The absence of fever, the want of thirst, the slight functional disturbance, the quiescence of the sympathies, will generally establish the neuralgic character of the pain. Should the affection have continued any time, the absence of emaciation is a most certain criterion of the neuralgic nature of the disease. Inflammation, especially chronic inflammation, if unattended by febrile symptoms, is always accompanied with emaciation. This circumstance will enable you pretty confidently to establish the diagnosis between the two diseases.

As to some of the visceral neuralgia, it is doubtful whether they are to be referred to the ganglionic apparatus, which furnishes the internal organs so abundantly with the nerves, or to the spinal apparatus which is in direct communication with the ganglionic, and through it with the viscera, or to both. As yet we do not know the relations maintained between these two important apparatus of the nervous system, and in the absence of positive facts we may as well abstain from hypothesis. It is a mooted point at this time, with physiologists, whether the ganglionic centres and nerves are endowed with sensibility. Should the absence of this function be established, then the neuralgias of course would belong wholly to the spinal centres and nerves. In many cases it would appear that the primary cause of the neuralgic disease is seated in the intestinal, or gastric surface, supplied with ganglionic nerves. The impression thus made on the ganglionic system would then appear to be transmitted to the spinal centres of sensibility, and thus excite the neuralgic pain.

As the external neuralgia the internal are fixed, or wandering; partial, or local, or general. We have them sometimes localized for years in one particular organ, at other times changing from day to day, one day in the head, another in the heart, another in the stomach, and so on.

A very common seat of neuralgic pain, which appears to be internal, is in the cardiac region, or its vicinity; sometimes a little lower, or to one side of it. It continues for years, but never involves the health of the patient, if not improperly treated. I have been consulted in fifty or more cases of it. I have never known it in males on the right side. It often gives rise to a great uneasiness; the individual is apprehensive of the approach of consumption, or disease ot the heart, or some similar affection. I was once consulted by a gentleman who had been sent three successive winters to the South by his physician, who apprehended that he was threatened with consumption. He was referred to me by his attendant physician to ascertain the state of his lungs. Still apprehending consumption he had been advised to seek again the genial clime of the Southern States. As his business suffered materially from his leaving the city, he objected to the arrangement unless absolutely necessary. I could not by a close investigation detect any physical signs of phthisis, and in their absence, as well as the want of the rational or general signs, I did not hesitate to pronounce the affection to be neuralgia. This occurred some eight or nine years since, and the gentleman, who is an intimate acquaintance at this time, has continued in perfect health, so that the diagnosis then made has been verified. I have seen many other patients of the same kind. A gentleman was about resigning a lucrative employment under government, supposing that he laboured under phthisis, and had but a short time to live. I diagnosticated neuralgia, and when I last heard from him, three years subsequently, his health remained unaffected.

The internal neuralgias often continue for years, producing great suffering, but not impairing the constitution. A gentleman consulted me sometime since whose case was of this kind. He was attacked with gastralgia two or three years since. This complaint not being understood, he was salivated, cupped, bled, purged, dieted; all without relief. Food had no influence upon him. No matter what he eat, a full diet, or a low diet, he suffered equally. He consulted me about two years ago. It was about the time that neuralgia was beginning to attract attention and to be understood. I took up his case with considerable care, and went through all the remedies with him; but with no avail. To this day he suffers as much as ever, the disease having baffled every method of treatment. Notwithstanding this long suffering, his general health is good; he has not lost flesh, nor is he affected in general appearance.

Having now laid before you the general character of neuralgia, the symptoms, nature, and treatment, will occupy our attention at our next meeting.

---

*Saturday*, *27th January*. Dr. Gibson presented to the class a girl, 25 years of age, with an ulcer on the upper lip. He described her case as follows: This girl has been generally healthy; she has had a sore on her upper lip for six months past. This first showed itself in the form of a small pimple upon the upper lip, with a yellow head. She pricked it with a pin, when it broke and discharged matter. She showed it to a Dispensary doctor, who upon seeing it, instantly whipped out a knife and cut a piece from the lip. Since then, it has been gradually growing worse, and an ulcer exists of some extent, as you see, which is spreading; it has already made a hole into the cheek. The girl has been in the hospital about two weeks, and has yet been placed under no particular plan of treatment. We can obtain no very satisfactory particulars of her previous history. As far as we can ascertain from her, she has not been affected with a syphilitic disorder, although she admits a slight discharge of a local character about six years ago. Many might be disposed to look upon this as a cancerous affection; but it is by no means well marked with the carcinomatous character. In the first place, it is upon the upper lip; and I have never seen but one or two, or, at most, three cases of carcinoma of the upper lip, while I have met with at least an hundred of the lower lip. I may say that it appears almost invariably in the *lower* lip, showing itself at first as a small shot-like tumour, deep in the substance of the lip, moveable at first, and afterwards becoming fixed to the surrounding parts. These swell and, finally, may ulcerate. Afterwards the glands under the jaw become enlarged. This you may look upon as a sure indication of carcinoma, distinguishing it particularly from lupus and syphilitic affections of the lip.

You may notice that the ulceration here has spread from the upper to the lower lip. In character, it most resembles lupus squamosus, but it wants some of the distinguishing marks of this affection. Lupus is a very peculiar disease; it was called by the older surgeons, (which name it still in a measure retains,) noli me tangere, or touch me not, from the belief that any application only aggravated it. This case differs from lupus squamosus, by wanting the scaly incrustations, which in that complaint, are to be seen generally about the alæ nasi, and the cartilaginous septum of the nose. Here, there is mere ulceration, with the ordinary redness attendant upon it. This, together with the swelling, gives the lip the appearance of a large red cherry. What then is it, if it be not lupus, or cancer, or a syphilitic ulcer? I am of opinion that it may have been a furunculus, or bile, which may appear in the cellular structure of any part of the body. It is, as you know, a conoidal tumour, with a central core; when this core comes out, a hollow remains, and ulceration is apt to extend to the surrounding parts. It cannot have been a carbuncle, for this spreads its ravages by sloughing and not by ulceration, and moreover does not usually attack the lip, but the shoulder, head, arms, and lower extremities. So peculiar too are its dark, black colour, and the burning, lancinating pain attending it, that it would easily have been recognized.

It is of course difficult to say whether this sore depends at all upon syphilitic taint; the statement of the girl that she has never laboured under any thing worse than a gonorrhea, and that six years ago, may be true or not. Pearson, and other British writers, have described syphilitic ulcers bearing a resemblance to this. It is not easy however, to determine them, with certainty, especially after the lapse of several months. But whatever this may be, whether lupus, or syphilis, or the result of a furuncle, the idea that entered the brain of the gentleman who cut it out, was a most unfortunate one. In the removal of the most trifling tumour, a good surgeon will always be cautious, and never venture to remove disease with the knife at first sight—as if fearful of losing a chance of an operation.

The treatment proper in this case will be to influence the constitution by general remedies such as sarsaparilla. Mercury would be here improper; to touch the gums by its use, would be to aggravate the symptoms of the disease. Any of the preparations of sarsaparilla may be employed; in this hospital, Marks' Extract is generally used. The best local applications will be mild poultices; that of the bark of the slippery elm is particularly soothing and relaxing. No operation could here do any good, you might cut away the whole lip, and the ulceration would still return. Even if an operation were indicated, I should hesitate to perform it upon this girl. So very nervous and irritable is she, that it would be apt to fail. I could induce her to enter the operating-room, only by a solemn assurance that I would not touch her with the knife.

The case I next present to you is that of a respectable old woman who has been the mother of nine children; you will not be surprised then to find her breast, or uterus, out of order. She is 78 years of age, although she does not look more than 60. The swelling in the breast she has had for nearly two years. It is you see in the left breast, from which she tells us she was most in the habit of suckling her children. This is a probable reason for its having taken on disease.

This is not as a case of carcinoma, although it might be very readily mistaken for it: it is merely a diseased state of the cellular tissue and surrounding texture. If it were genuine scirrhus, the nipple would by this time have been curved in, owing to a condensation and retraction of the parts, and adhesions to the pectoral muscle. The glands of the arm-pit would probably have been also affected, which is not the case. In cancer you have severe lancinating pains, as from the pricks of pins or needles, or as if some animal were tearing the parts inside. I have known negro women to imagine that a ground puppy, as they term it, was clawing away at their insides, and of negro doctors persuading them that they had cured them by taking it out. All symptoms of this sort are wanting in the woman before you.

A young surgeon might be tempted to operate in this case. But even if it were carcinoma, I should deem an operation of very doubtful policy, at this woman's advanced age. The operation for the removal of cancer is, under the most favourable circumstances, one of considerable uncertainty and risk, and under unfavourable circumstances, rarely of permanent advantage. It may save a few years of life; but I doubt whether it would lengthen the span of this old lady's years. If she were thirty-eight instead of seventy-eight, and really had cancer, there would be some propriety in an operation. I look upon the case as enlargement of the mamma, from disorder of the cellular membrane. The breast as well as the uterus, with which it has a strong sympathy, has been overworked, and the consequence has been an inordinate development of cellular structure. There is an absence of all the genuine characteristics of scirrhus.

The long period which has elapsed since the flow of catamenia, is an argument against the existence of carcinoma. It is most apt to show itself, in parts which have lately ceased to perform the functions for which nature designed them. Thus a blow on the testicle or penis, at the age of fifty or sixty, will, in a bad constitution, be apt to degenerate into a cancerous affection. In women, the uterus, when it has ceased to bear children and discharge the catamenia, is similarly liable to take upon itself diseased action. So it is with the breast.

[Dr. Gibson next made some general remarks on the subject of stricture of the urethra, which are not reported for want of room. The surgical lectures at the Pennsylvania Hospital are postponed for a similar reason.]

Dr. Gibson concluded as follows: with this lecture, gentlemen, terminates my present clinical course. Before parting with you, I have one observation to make. You have perhaps noticed that I have had but few operations to present to you during my tour of duty. I may be allowed to instance this circumstance, occurring as it does in an hospital where we have generally, upwards of four hundred patients under care and which is certainly one of the best clinical establishments in the United States, as a striking illustration of the improvement of surgical science. As surgical knowledge accumulates, the mania for *cutting* disappears. It is *medical* surgery, gentlemen, that should occupy your attention. Operative surgery is diminishing daily, and will be yet more diminished. You will, or ought to, have few, very few, operations to perform. It requires more skill to cure than to cut. A *mere* cutter needs no more brain than a pigeon or tom-tit; his whole sense is at his finger ends. I do not mean to say, however, that *operations* are never necessary; on the contrary, many diseases cannot be cured without them; but that the man who is best prepared to decide when an operation is required, and who, at the same time, performs it, coolly, deliberately, without affectation, display, or flourish, is to be looked upon, in every respect, as the best surgeon.

Of the cases we have had under notice, at former lectures, those of sloughing ulcer and of chronic mortification terminated fatally as I predicted. The man, whose jaw I removed, is, for the present, relieved, the wound having cicatrized. The final result time will disclose.

---

### NEURALGIA.

Dr. Jackson followed Dr. Gibson at 11½ o'clock.

When I last met you I made some general remarks upon neuralgia. I shall continue the subject to-day. I shall not however be able to present to you any additional specimens of the disease, as unfortunately, or perhaps I ought to say fortunately for the patients, we have just at this moment no others in the house laboring under the disease, than those that have already been before you. Yesterday I could have exhibited to you in my own person a very excellent specimen of an attack, but to-day I am happily free from it.

I mentioned to you at our last meeting the various forms and locations of neuralgia; of its being internal and external; general and local, at one time involving the whole, or half of the body, at another occupying an inch to an inch and a half of a nerve only; occasionally limited to a single point which might be covered with the end of a fine needle or pin, yet occasioning the most excruciating torments. It occurs too in the dura mater, and perhaps too in the arachnoid. None indeed of the internal organs are exempt from it. The liver, lungs, heart, spleen, uterus, bladder, stomach, and intestines, are all equally subject to it. Some of the most intractable forms of the disease, which I have ever witnessed, have occurred in the urethra of the female. In the female too the extremity of the os cocygis is not an uncommon location of the disease; the point of pain can in this case be covered with the end of the finger, yet it is productive of the intensest torments. Neuralgic pain is sometimes persistent, remaining in one spot during its whole course, or it is erratic, every part of the body being affected in succession. The attacks are frequently irregular, coming on at no definite periods. Again it is intermittent, or paroxysmal, assuming the quotidian, tertian, or quartan forms. This is very common about the external angle of the eye, and face, where the branches of the fifth pair of nerves are involved.

There are two different and distinct forms of neuralgia. The one is inflammatory; positive inflammation; the nervous cord, like all the other tissues, being liable to inflammation. In the other form there is mere functional derangement, without structural lesion. We can understand perfectly well how the first form is produced. Inflammation may occur in the nerve, or neurilema, and disorder of function be produced, precisely as inflammation causes disorder of function in any other organ in which it is seated. Or pressure may be produced between the nervous fibrils and the neurilema, or thickening of the neurilema, by the formation of small tumours, osseous spicula, or other morbid deposits.

In the explanation of functional derangement we encounter greater difficulty. We do not yet know in what sensation consists; we do not rightly comprehend the physiological phenomena; we cannot therefore with any certainty indicate the pathological deviations. There are two modifications of functional neuralgia: the one consisting in simple exaltation of sensibility, as in the case of the young girl you saw last Saturday, who could not endure the slightest touch, without experiencing the acutest pain. The other variety is a perversion of sensibility, as where there exists intense pain in a part without an exciting cause.

A diagnosis is to be established between inflammatory neuralgia, and neuralgia arising from mere functional derangement. This is generally easily accomplished. The former succeeds very often to rheumatism, from exposure to cold, and injuries. We should in these instances learn the history of the case, and inquire into the causes. Inflammatory neuralgia occurs too after operations, and especially amputations. The extremity of the nerve becomes inflamed either from the wound or exposure to the action of the air. In such cases it is found to be thickened, enlarged into a tumour, or otherwise changed in its structure. Mr. Swan recommends, in order to avoid the danger of producing this distressing accident, that the nerve be cut off above the wound. Instances are related where repeated amputations have been performed to relieve the patient. A very remarkable case has been lately related by Mr. Mayo. A girl submitted to several separate amputations to be relieved from the excruciating agony she endured from neuralgic pain. The limb was finally removed at the hip-joint. When I saw the last account, the disease had not yet returned; what the event will be it is impossible to prophecy. The nerve in this case was found diseased.

Inflammation of a nerve often succeeds the operation of bleeding from a slight puncture of the nerve. I have seen several cases where the use of the arm was lost from this accident. The pain extends over the whole arm, rigidity and stiffness of the finger joints occur, and in some cases swelling with heat. Mr. Mayo asserts that if the nerve be divided early, before the wound is cicatrized and the whole arm involved, that the patient will recover, but that the operation, after the inflammation has extended throughout the nerve, is useless. Small tubercles of an inflammatory character are formed sometimes on nerves, or they excite inflammation in the nerve. They are exceedingly painful. Mr. Wood mentions them under the name of painful tubercles, and Sir Everard Home also relates several instances that fell under his notice.

These are cases of inflammatory neuralgia; I have met with a number of instances of the affection, and I have succeeded in establishing the diagnosis by the effects of pressing upon the nerve. If in functional neuralgia pressure be made upon a nerve, the pain is relieved. Thus in ischiatic neuralgia the application of firm pressure gives often instant ease. In my own case the application of a ligature on the limb during an attack diminishes the pain very much; it does not relieve me entirely, but procures a diminution of the intensity of suffering; it renders it bearable. Now the contrary obtains in inflammatory neuralgia. Pressure along the course of the inflamed nerve aggravates the pain, and renders it insupportable to the patient. The next point of diagnosis to which I shall call your attention is the distinction between the internal or visceral neuralgia and inflammations. This sometimes proves perplexing and is not without difficulty. Mistakes are by no means uncommon especially with those who regard pain as always indicating inflammation. In external neuralgia it is easily accomplished; none of the obvious signs of inflammation are present; there is no change in the appearances of the part; no heat, no swelling, no redness, while in inflammation these symptoms are always attendant. The internal neuralgias, in which these symptoms are withdrawn from observation, offer great difficulties, and hence are liable to be confounded with affections demanding for their cure directly opposite modes of treatment. We must rely in a great measure on the causes; these will direct and assist us materially. The general symptoms too lend us important aid in determining between the internal phlegmasiæ and the neuralgias. Thus in an attack of pleurisy you would have with pain, cough, increased pulse and febrile symptoms. Not so in pleurodynia; you would have no general affection of the system, and there occurs none of the peculiar deposits in the urine so common in the affections of the chest.

The discrimination is however often difficult, especially in persons of a sanguine temperament, and phlethoric habit, for though not a common occurrence, yet I have seen the affection in such individuals. I remember being called to a person of fine, robust constitution, phlethoric habit, and one in whom neuralgia would not have been looked for. Whilst heated he had exposed himself to cold. He had some cough, violent pain in the left side, increased by inspiration, a strong, full pulse, and flushed face. The chest was resonant, but the respiratory sound was scarcely audible. Here the causes and general symptoms indicated the presence of inflammation, and I commenced the treatment accordingly. But depletion general and local gave no relief, and the result of the treatment first intimated the true nature of the disease, and convinced me that I was in error. Quinia with narcotics relieved my patient in twenty four hours. The treatment often in very doubtful cases gives the true character of the disease. In neuralgia the antiphlogistic treatment increases the severity of the symptoms.

In neuralgias of the chest the physical signs (auscultation and percussion) are mainly to be relied on in assisting our diagnosis. It is to be remarked that where the pain is very acute, the movements of the chest are feeble, and vesicular respiration is so faint as not to be detected. This might lead to the belief that engorgement and obstruction existed, but percussion will at once decide the question. The entire absence or lightness of any febrile movement, so little corresponding with the intensity of the pain, or presence of a violent inflammation, will also assist in deciding the point.

From the facts thus determined we may then conclude that it is not a pleurisy or pneumonia, but a neuralgic affection of the chest. Should there still exist doubt, take blood, and if no relief be obtained, but the symptoms are increased, then change your course, give quinine, combine it with opiates, as aconite, belladonna, hyoscyamus, &c. Give too some warm, sudorific stimulating potion, with the view of producing diffusion—a fluctionary movement to the surface.

The different influence of excitants will also determine the difficulty. In neuralgia the stimulants relieve; in the inflammations, on the other hand, they aggravate all the symptoms.

Having now pointed out to you the various forms of the disease, and exhibited to you several interesting specimens illustrative of these, and having attempted to show you the diagnosis between the different varieties of neuralgia, and between neuralgia and inflammation, I shall proceed to speak of the treatment, and shall first call your attention to the inflammatory form of the affection.

In this form of the disease the patient is to be placed on a light mild diet, and a modified antiphlogistic treatment is to be pursued. Some cases will require general depletion, but it is from local depletion that the greatest benefit is to be derived. Leeches and cups are to be placed along the course of the inflamed nerve immediately above it. Cups or leeches are to be applied on the spine. Laxatives, purgatives and alteratives are to be resorted to. The purgative of colchicum wine and magnesia is well adapted to this state; and if the pain be severe the combination of colchicum and black drop is to be given at night in full doses. I have found great benefit to result from warm fomentations and poultices. The whole limb is to be enveloped in them and they are to be renewed when they become cold.

Functional neuralgia is more difficult to manage. We do not know in what sensibility consists, how it is generated, or the modes of its derangements. All that we are certain of is, that sensibility originates in the cerebro-spinal system. We should never lose sight of this, and our treatment should always be directed with a view of operating on these centres. Now, in our last lecture, I told you of particular centres presiding over particular portions of the body, and I explained to you the segmentation of the nervous system, as corresponding to regions of the body. As I then mentioned, we have neuralgia sometimes a general affection, sometimes localized down to a point. Some writers seem disposed to think that all the conditions may be referred to the spinal marrow. I am myself much inclined to doubt this; for, in the local neuralgia, where it is persistent and limited to one spot, the applications to the spine are of no utility whatever. Now every nerve has to a degree a special and peculiar sensibility of its own. In the wandering neuralgia, the pain varies with its situation, but it is always the same in the same nerve. This I know in my own case is an invariable rule; the character of the pain varies, as it shifts. Hence I am inclined to infer, that it cannot all originate from one source, and that the nerves themselves must exercise some specific influence.

We have, as I told you, two forms of modification of increased sensibility; simple increase or exaltation, and perversion of sensibility. In the case of the girl, shown to you at our last meeting, a dull or sharp body each excited as much torture as the other. A mere touch with the finger gave intolerable pain, as you saw when I placed my fingers on her. This was an example of simple augmentation of sensibility. In other cases, there is perversion of sensibility: the sensation of constant burning and itching, in various parts of the body, exists without the agency of any cause, capable of producing these feelings.

We have the same perversion occurring in special sensibility: as, in the appetite, taste, sight, hearing. Some persons hear noises which have no existence; others again have hallucination of vision, seeing objects with perfect distinctness that are not present.

(To be Concluded in Number 5.)

### CLINICAL REPORTS.

PENNSYLVANIA HOSPITAL.

